# Lung Ultrasound in the Neonatal Intensive Care Unit: Does It Impact Clinical Care?

**DOI:** 10.3390/children8121098

**Published:** 2021-11-29

**Authors:** J. Lauren Ruoss, Catalina Bazacliu, Nicole Cacho, Daniele De Luca

**Affiliations:** 1 Division of Neonatology, University of Florida, Gainesville, FL 32610, USA; cbazacliu@ufl.edu (C.B.); nicole.cacho@peds.ufl.edu (N.C.); 2 Division of Pediatrics and Neonatal Critical Care, A. Béclère Medical Center, Paris Saclay University Hospitals, APHP, 94270 Paris, France; daniele.deluca@aphp.fr

**Keywords:** lung ultrasound, point-of-care ultrasound, neonatal respiratory pathologies, respiratory failure

## Abstract

A neonatal point-of-care ultrasound has multiple applications, but its use has been limited in neonatal intensive care units in the Unites States. An increasing body of evidence suggests that lung ultrasound performed by the neonatologist, at the bedside, is reliable and accurate in differentiating neonatal respiratory conditions, predicting morbidity, and guiding invasive interventions. Recent research has shown that a lung ultrasound can assist the clinician in accurately identifying and managing conditions such as respiratory distress syndrome, transient tachypnea of the newborn, and bronchopulmonary dysplasia. In this review, we discuss basic lung ultrasound terminology, evidence for applications of neonatal lung ultrasound, and its use as a diagnostic and predictive tool for common neonatal respiratory pathologies.

## 1. Introduction

Neonatal point-of-care ultrasound (POCUS) was first described in the 1960s, with increasing applications being reported over the past decade [1,2,3]. Neonatal lung ultrasound (LUS) is used in emergent situations, differentiating neonatal respiratory pathologies, and predicting neonatal morbidity [2]. LUS can be brought to the bedside of the fragile neonate, used serially, and does not expose the neonate to ionizing radiation [1,4]. Functional and descriptive applications make it a high-fidelity tool to aid in distinguishing the various causes of neonatal respiratory failure and to guide in management [2,5]. LUS also has increased sensitivity and specificity in comparison with an X-ray for the detection of respiratory pathologies (e.g., pneumothorax and pleural effusions) and can be utilized to monitor progress of clinical pathologies [6]. The neonatologist–performed LUS has the advantage of being immediately interpreted by those caring for the neonate, potentially leading to more accurate diagnosis and timely therapeutic intervention [5]. Neonatal POCUS has a multitude of applications and benefits, but its widespread use is limited by the lack of a standardized education curricula and the difficulty of implementing effective quality assurance programs. While the European resuscitation guidelines now recommend utilization of LUS for the confirmation of endotracheal tube placement (ETT), cardiac tamponade, pneumothorax, and pneumonia, its application is not routinely taught in modern neonatology training around the world [7]. This review focuses on the descriptive use of neonatal LUS in evaluating common neonatal respiratory pathologies and its functional use in management and prediction of neonatal respiratory morbidities.

### 1.1. Performing Neonatal Lung Ultrasound

LUS is especially useful in neonatology due to the neonatal chest anatomy, unique ossification of the thorax, and respiratory pathologies [1,8]. A linear high frequency probe, typically >10 MHz, is commonly used in neonatal LUS [1,9]. The high frequency probe is utilized due to its high resolution with a superficial depth of penetration. Higher frequency probes, such as micro-linear with a hockey stick shape, are commonly used for vascular access but are also useful in preterm neonates due to their small footprint. Moreover, different probe types can be used by an experienced operator to recognize the main LUS semiology [10]. The common modes used in LUS are 2D B-mode (brightness) and M-mode (motion), with an expanded use of a color doppler to evaluate blood flow [9]. The neonatal LUS is typically performed supine with direct identification of subcutaneous tissue, ribs, pleural line, and lung sliding with indirect evaluation of lung tissue [11].

#### Lung Ultrasound Score

LUS scores provide a standardized approach for a semi-quantitative assessment of neonatal lung pathology and the progression of disease [2,11,12,13,14]. Neonatal LUS score is commonly calculated by six chest areas that interrogate the anterior and lateral chest. In the first days after birth, there is little distinction between the dependent and non-dependent lung zones, where gravity has yet to play a significant role [15]. Each zone is assigned a score of 0 to 3, with total score between 0 and 18. The number of B-lines and subpleural consolidations per lung zone help distinguish different neonatal respiratory pathologies [11]. This approach to LUS is reproducible, with a high inter-observer agreement [15] and has the potential to be used to guide management and risk stratify neonates [7]. LUS score calculations on more advanced chest segmentation (10 or 12 lung zones) have been proposed after the first days after birth but may need more refining, as they did not result in a more accurate prediction and management of bronchopulmonary dysplasia (BPD) than the LUS score on the six zones [16].

### 1.2. Lung Ultrasound Terminology

**Figure 1:**
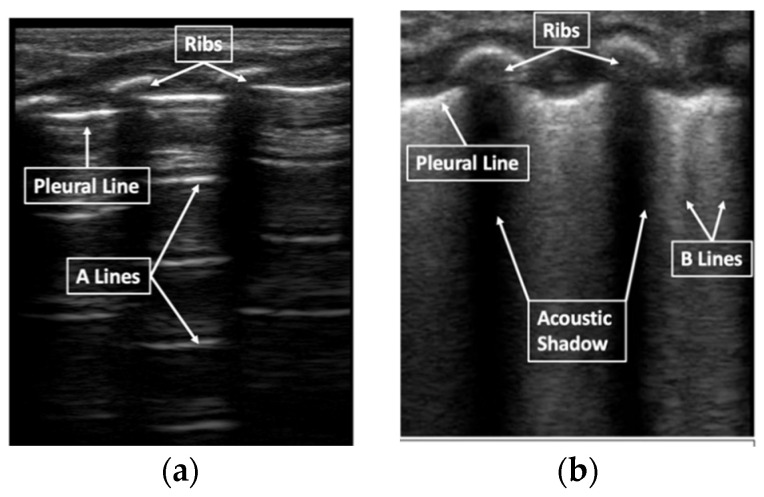
Lung ultrasound artifacts. B-model normal lung (**a**) B-model respiratory distress syndrome (**b**).

**Figure 2:**
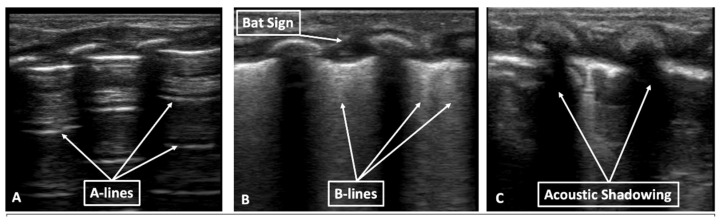
Lung ultrasound in B-mode. (**A**) A-lines, reverberation artifact from pleural line (**B**) B-lines, reverberation artifact from interstitial edema; (**C**) acoustic shadowing from the ribs.

Neonatal LUS is an evaluation and interpretation of normal lung artifacts. LUS identifies the subcutaneous tissue, muscle, ribs, and pleural line (visceral and parietal pleura interface) (Figure 1). Normal LUS artifacts include reverberation artifacts (A-lines), comet tail artifacts (B-lines), and acoustic shadowing artifacts (rib shadowing). Specific LUS terminology is used to describe the artifacts commonly seen in neonatal LUS (Figure 2).

#### 1.2.1. Pleural Line

The pleural line represents the visceral and parietal pleura and is demonstrated by a hyperechoic horizontal line. The pleural line is visible in neonates with and without respiratory pathology and becomes apparent following the first four breaths after birth [17]. The bat sign is exhibited by visualization of the pleural line and two adjacent ribs (Figure 1 and Figure 2). In a normal lung, the pleural line is thin and regular, while it can appear thickened and coarse in the setting of respiratory pathology.

#### 1.2.2. A-Lines

A-lines are reverberation artifacts from the reflective pleural line and are demonstrated as horizontal, equally spaced, hyperechoic lines that extend deep into the 2D image (Figure 2). Acoustic rib shadowing represents an artifact from the ribs, demonstrated by an anechoic area below the ribs that extend deep into the 2D image and disrupt the A-lines.

#### 1.2.3. B-Lines

B-lines or comet tail artifacts represent reverberation artifacts from interstitial edema or scarring of the interlobar septae in the lung parenchyma [1,8]. B-lines are represented as vertical lines extending from the pleural line to the edge of the screen and can be seen moving with lung sliding (Figure 2, Supplementary Materials Video 1A). B-lines can be seen in a normal neonatal lung, decreasing in number over time, and can be persistent in healthy newborns up to 6 months of age [18,19]. Compact coalesced B-lines, also known as “white lung”, are visualized in severe alveolar interstitial syndrome.

#### 1.2.4. Lung Sliding

**Figure 3:**
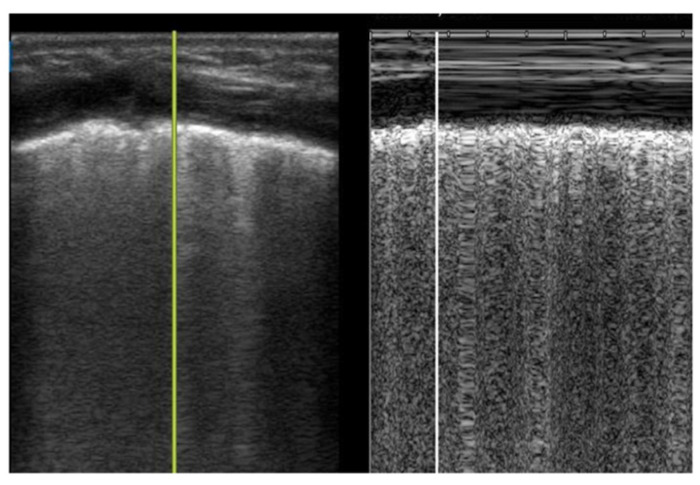
Lung sliding in M-mode. Very low birthweight infant with respiratory distress syndrome with normal lung sliding (seashore sign).

Lung sliding represents the sliding of the visceral and parietal pleura with respiration and can be visualized in B-mode or M-mode. In B-mode, lung sliding is described as the movement of ants marching along the pleural line with respiration (Supplementary Materials Video 1A,B). In M-mode, lung sliding appears as the seashore sign, where the static structures above the pleural line are the sea, and the movement below the pleural line creates the granularity of a sandy shore (Figure 3). Absent lung sliding can be encountered in any condition that affects the interpleural space, including pneumothorax, pleuropneumonia, complete atelectasis, and severe hyperinflation, as visualized through foreign body aspiration [20].

## 2. Applications of Neonatal Lung Ultrasound

Neonatal LUS has a multitude of applications including rapid assessment of the decompensating neonate, procedural guidance, and predication of morbidity. The POCUS working group of the European Society of Paediatric and Neonatal Intensive Care published guidelines for the use of neonatal POCUS in 2020 [21]. This group stated that there was reasonable evidence for neonatal LUS to distinguish respiratory distress syndrome (RDS) vs. the transient tachypnea of newborn (TTN), detect pneumothorax, and pleural effusions, and guide thoracentesis (level B). This statement should be taken into context with recognition of limitations of LUS, varying ultrasound features of these pathologies, and operator training. The evidence for evaluating pulmonary edema and detecting anesthesia-induced atelectasis was weaker (level C). Several algorithms have been proposed for neonatal LUS, including the assessment of life threatening emergencies [2,22], neonatal respiratory pathologies algorithm [8], and the neonatal LUS protocol [23,24]. The SAFE-R protocol was designed for use in the decompensating neonate and includes the evaluation of cardiac tamponade, pneumothorax, pleural effusion, and myocardial function [22]. These algorithms need further study to evaluate probe selection, patient selection, and management strategies.

### 2.1. Pleural Effusion

**Figure 4:**
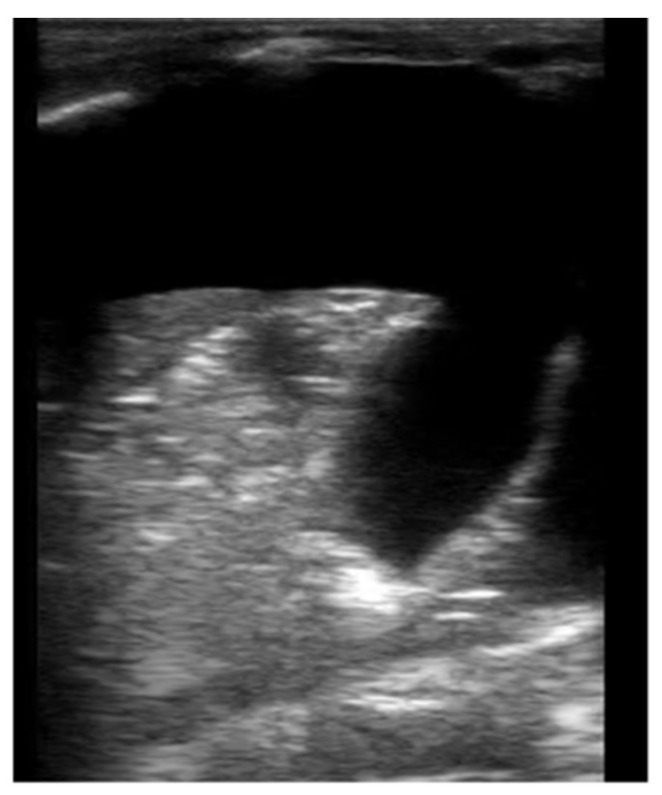
Large pleural effusion in B-mode. Large pleural effusion with septation and complete atelectasis of the lung.

Neonatal LUS can detect small volume effusions and can be used to guide thoracentesis [25]. In B-mode, the transudate fluid is anechoic, and the lung parenchyma can be visualized as hepatization of the lung (Figure 4, Supplementary Materials Video 2). In M-mode, the sinusoid sign can sometimes be visualized where the visceral line moves towards the pleural line with respiration. Color doppler can also be used to evaluate echogenic vs. solid collections within the effusion, however this is not commonly used in neonatal LUS.

### 2.2. Pneumothorax

LUS detects pneumothorax with high sensitivity and specificity (96.7%, 100%) and is reproducible [26]. In a recent systematic review and meta-analysis, LUS was superior to a chest X-ray in diagnosing pneumothorax [27]. An international multicenter study demonstrated that LUS can effectively and safely detect life-threatening pneumothoraces and guide chest drainage without the need of chest X-rays [28]. A pneumothorax is demonstrated by an absence of lung sliding and an absence of B-lines, however it is important to note that any pathology that disrupts the visceral and parietal interface will have absent lung sliding. Identification of the area where the parietal and visceral pleura separate is called the lung point [27], though this finding may be absent in a large tension pneumothorax [29,30].

### 2.3. Atelectasis

**Figure 5:**
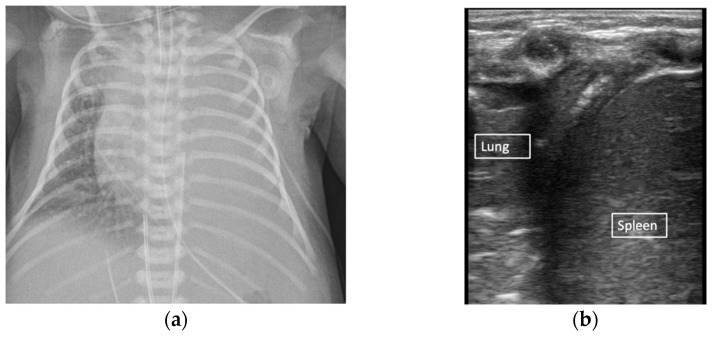
Effusion vs complete atelectasis. Large effusion suspected on chest X-ray (**a**). B-mode demonstrating complete atelectasis of the left lung without large pleural effusion (**b**).

Atelectasis is demonstrated as a consolidation with the anechoic border and a disruption of A-lines [8,31]. Complete atelectasis can cause an absence of lung sliding and hepatization of the lung on ultrasound [1,31]. Lung pulse can also be observed in severe atelectasis, where the atelectatic lung appears to be pulsating with the beats of the heart [31]. Atelectasis can present as static air bronchograms in early stages vs. pneumonia, which is demonstrated as dynamic air bronchograms that move with respiration [32,33]. In clinical practice, it is difficult to discern static vs. dynamic air bronchograms, making the diagnosis of atelectasis vs. pneumonia a continued challenge. Complete atelectasis vs. pleural effusion can be difficult to distinguish on a chest X-ray, but can be easily identified on LUS in real time (Figure 5, Supplementary Materials Video 3). 

### 2.4. Transient Tachypnea of the Newborn

TTN presents as respiratory distress after birth secondary to delayed fetal fluid clearance. TTN is distinguished from RDS based on the identification, number of, and location of B-lines [34,35]. TTN presents as bilaterally symmetric B-line distribution with a regular pleural line. The double lung point, the area between the upper and lower lung fields where spaced-out B-lines are next to confluent B-lines, has high specificity but low sensitivity for TTN and is not needed for the diagnosis [30,34,35]. The most important thing is to recall that TTN is an “extrinsic” edema, and therefore appears as such, though with spared lung areas with A-lines (which is not the case of RDS due to primary surfactant deficiency) or consolidations. A meta-analysis performed in 2021, including over 1500 neonates, concluded that LUS has high specificity and sensitivity for diagnosing TTN [36]. A multi-centered trial evaluated neonates with TTN and found that 47.6% had the double lung point sign and all had a regular pleural line without consolidation on LUS [34]. The LUS score has also been shown to predict the need for intubation in neonates with respiratory failure on day 1 after birth [5], and has a high sensitivity, specificity, and positive predictive value (77.7%, 100%, 100%) for predicting the need for respiratory support. [37] Based on available data, LUS can be used to diagnose TTN and has been proposed as a modality to predict which neonates should be transferred to a higher level of care. Mixed RDS/TTN situations do exist when impaired lung fluid re-absorption is associated with relative surfactant deficiency but can be identified by LUS score [38].

### 2.5. Respiratory Distress Syndrome

**Figure 6:**
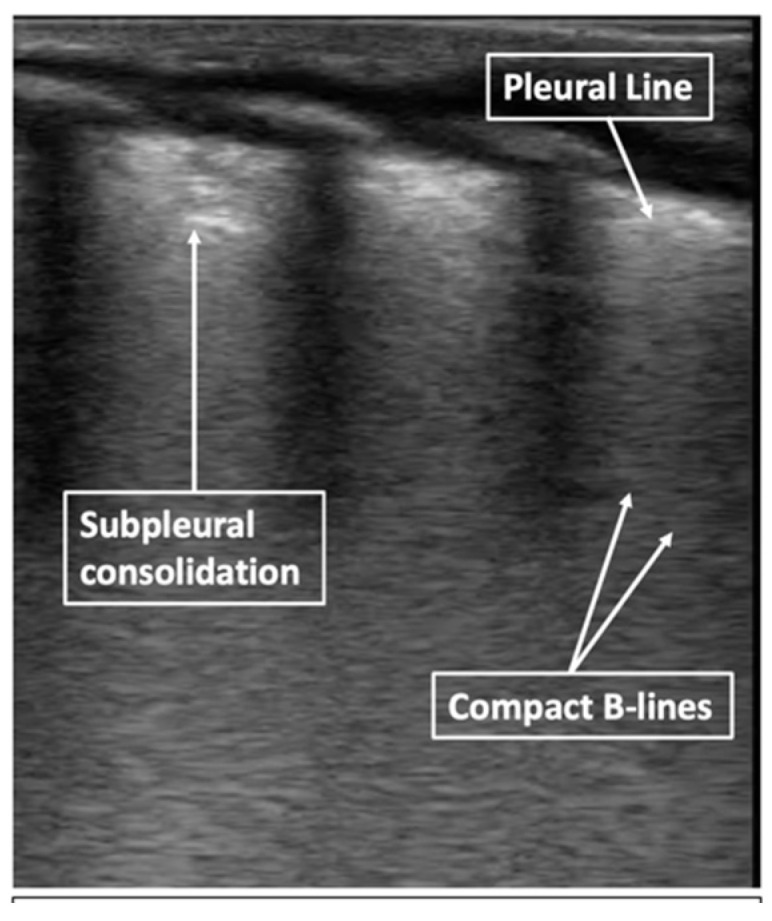
Severe RDS in B-mode. Compact B-lines (white lung), subpleural consolidation, thick and irregular pleural line.

RDS is a common reason for a neonatal intensive care unit (NICU) admission and occurs in neonates delivered prematurely or with impaired surfactant production. RDS presents similar to TTN with tachypnea and oxygen requirement after birth. Neonates with RDS have a thickened and coarse pleural line, irregular hyperechoic subpleural consolidations, and numerous B-lines. In severe RDS, the lung can have a white-out appearance where there is a confluence of B-lines (Figure 6). Copetti et al. demonstrated that alveolar interstitial syndrome (white lung), in an abnormal pleural line without areas of a normal-appearing lung, had a 100% sensitivity and specificity for RDS [39]. A recent meta-analysis of neonatal LUS concluded that LUS has significant diagnostic accuracy, with an area under the curve (AUC) of 0.99 when diagnosing RDS [40]. LUS can be reliably used to diagnose RDS in the neonate and may be able to predict need for intervention. 

#### Predicting the Need for Intervention with Respiratory Distress Syndrome

Neonatal LUS has been shown to predict the need for surfactant and mechanical ventilation. Raimondi’s group demonstrated that the diagnosis of the “white lung” in neonatal respiratory distress predicted the need for intubation with high sensitivity and specificity (88.9%, 100%) [41]. Brat et al. then demonstrated that the LUS score performed in the first several hours after birth correlated with oxygen indices and predicted the need for surfactant [15]. Badurdeen et al. and De Martino et al. went further to show that LUS could predict the need for surfactant with more accuracy than fraction inspired oxygen (FiO_2_) [42,43]. In 2021, Raimondi et al. published a multicenter pragmatic trial that showed that LUS score predicted the need for surfactant (AUC 0.86), led to earlier surfactant administration, and decreased oxygen requirement in comparison to utilizing FiO_2_ requirement for surfactant administration [44]. Echography-guided Surfactant THERapy (ESTHER) utilizes the LUS score to guide surfactant administration and results in an earlier administration of surfactant and a decrease in the duration of invasive ventilation without increasing cost [45,46,47].

### 2.6. Bronchopulmonary Dysplasia

BPD is a common complication of prematurity and is defined as the need for respiratory support and/or oxygen supplementation at 36 weeks corrected gestation. BPD is demonstrated on LUS by a thickened coarse pleural line with subpleural consolidations and B-lines. The B-lines can be present as scattered or diffuse, depending on the amount of interlobar septae scarring and interstitial edema. As previously mentioned, the LUS score has been utilized to diagnose the severity of BPD [48] and to guide management, including the use of diuretics [14]. More research is needed regarding the utility of LUS to predict morbidity associated with BPD, including readmission and the need for bronchodilators, and to differentiate severe and mild BPD.

#### Predicting the Development of Bronchopulmonary Dysplasia

BPD carries significant morbidity in the neonatal population, and it is difficult to predict which infants will develop this outcome. Neonatal LUS has been evaluated for its use in predicting BPD, which would enable an earlier individualized approach to management. LUS patterns in the first four weeks after birth have been found to be a reliable predicter of BPD [13,44,49,50]. LUS has been shown to have a moderate accuracy at predicting BPD even as early as the third day after birth [14]. In a multicentered international longitudinal cohort study, the LUS score on day 7 and 14 correlated with oxygenation indices and, when adjusted by gestation and sex, predicted BPD (*p* < 0.0001) [16]. In a recently published prospective diagnostic cohort study, LUS was done on day 3, 7, and 14 in 152 infants less than 29 weeks gestation [49]. The LUS score was higher in infants who later developed BPD than in infants without BPD on all time points. A LUS score > 10 on day 7 had the highest sensitivity and specificity for predicting BPD. Utilization of the LUS score in the first weeks of life has vast applications as it can provide neonatologists with the knowledge needed to individualize therapy and design randomized control trials targeting a subgroup of neonates at risk for BPD. 

### 2.7. Broader Applications of Neoantal Lung Ultrasound

Neonatal LUS has been evaluated for broader applications including ETT position, vocal cord assessment, diaphragm abnormalities, and congenital pulmonary airway anomalies. LUS has also been studied for its use in other special circumstances, including lung recruitment during anesthesia and the assessment of pulmonary congestion in the setting of congenital heart disease and shock, that are beyond the scope of this review [51,52]

#### 2.7.1. Pulmonary Hemorrhage and Meconium Aspiration

**Figure 7:**
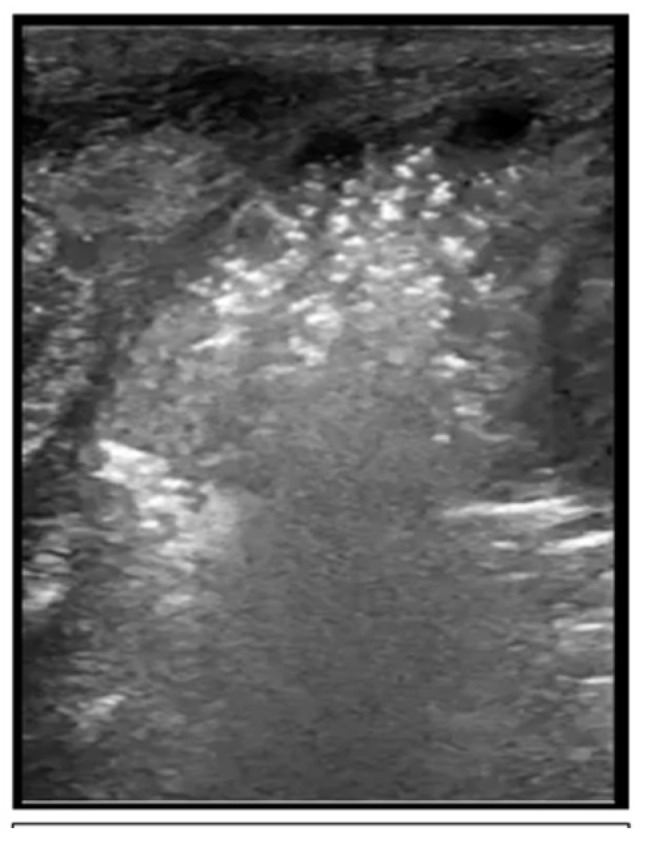
Pulmonary hemorrhage in B-mode.

Pulmonary hemorrhage (PH) is secondary to pulmonary venous congestion and pulmonary capillary blood extravasation into the alveolar space. Although this complication is associated with significant morbidity, few papers have reported the use of LUS for the evaluation of PH in neonates [30,53,54]. PH on LUS is demonstrated as a large consolidation with a shred sign (irregular or shredded border), air bronchograms, and disruption of A-lines [54]. Clinical context is vital as pneumonia has similar features on LUS and is difficult to distinguish from PH [53] (Figure 7). 

Meconium Aspiration Syndrome (MAS) is caused by in utero aspiration of meconium into the airways. It is encountered in neonates at or close to term gestation and is potentially lethal. Piastra et al. and Liu et al. described characteristics of MAS in LUS as pleural anomalies with subpleural consolidations, lung consolidations with a shred sign, air bronchograms, and consolidated B-lines [55,56]. Those areas alternate with less involved or spared areas, with differing patterns of distribution as a result of the disease heterogeneity. While Corsini et al. found 100% agreement between LUS and chest X-ray diagnosis in MAS, more data is needed for its recommendation in routine use [57]. Although LUS has been used to describe the characteristics of MAS, clinical correlation with the neonatal delivery history and clinical picture is needed.

#### 2.7.2. Endotracheal Tube and Vocal Cord Assessment

The ETT position by LUS has gained interest as a quick assessment of the ETT position in the decompensating neonate is needed and numerous X-ray are required for ETT positioning in the NICU. A small number of feasibility studies have demonstrated that LUS could be used for assessment of ETT positioning in the trachea. The position of the ETT was determined using either a phase array probe, in a high parasternal view, or a linear probe, in a midsagittal view. The position of the ETT was determined by the distance between the end of the ETT and the apex of the aortic arch [58] or the distance between ETT and the superior aspect of the right pulmonary artery [59]. Chowdhry et al. reported a high concordance between X-ray and ultrasound assessment of the depth of the ETT [58], while another group reported use of LUS for immediate confirmation of ETT placement during neonatal resuscitation using a linear probe in transverse position [60]. The use of LUS for ETT positioning is promising for its ability to decrease the time for assessment in the unstable neonate. However, more research is needed prior to routine use [21].

Another potential application of ultrasounds is the assessment of vocal cord function. Lee et al. showed that a laryngeal ultrasound could identify vocal cord paresis in 52 newborns who underwent aortic arch repair in a Norwood-type procedure with a high sensitivity and specificity when compared to flexible fiber optic nasoendoscopy [61]. Visualization of the vocal cord was performed with a high frequency linear hockey stick probe, in transverse position, over the midline of the neck [61].

#### 2.7.3. Diaphragm and Developmental Pulmonary Anomalies

An evaluation of the diaphragm and developmental of pulmonary anomalies could prove useful in risk stratification and individualizing management. Utilization of LUS for the evaluation of diaphragm health, including extubation readiness, has been reported in adults and pediatric critical care [62,63]. A recently published study was the first to report neonatal LUS and diaphragmatic shortening fraction, which has been used in adults to assess diaphragm function. This group found that the diaphragmatic shortening fraction could be assessed in preterm and term neonates, which could expand the already broad utility of neonatal LUS [64]. While the fetal ultrasound is primarily used to evaluate developmental pulmonary anomalies (e.g., congenital pulmonary airway malformations—CPAMs) and congenital diaphragmatic hernias, few studies have reported on the utility of postnatal LUS [65,66]. A recent case series described the use of LUS in neonates with congenital diaphragmatic hernia, reporting specific LUS patterns for this pathology [67]. However, more research is needed, as there are varying phenotypes of congenital diaphragmatic hernia with significant morbidity. The ability to non-invasively evaluate severity, guide lung recruitment, and diuresis could impact outcomes [67]. LUS has also been proposed as a modality to follow asymptomatic CPAMs [68], and recent literature has reported common patterns of CPAMs in neonates [65]. As the application of LUS broadens, new studies are needed to assess its ability to replace other, more invasive, diagnostic methods.

## 3. Conclusions

Neonatal LUS is increasingly being used in neonatal intensive cares units around the world secondary to its broad applications. LUS is widely available, non-invasive, can be performed serially, and interpreted by the providers caring directly for the neonates. As highlighted in this review, neonatal LUS has numerous applications expanding well beyond the evaluation of the acute decompensating neonate. LUS can discern the difference between various common respiratory pathologies, evaluate for common causes of acute deterioration, and be used to predict severe morbidities. Despite its many uses, neonatal LUS has several limitations, including the need for quality assurance and support from outside services such as radiology. Proper training is needed, however; to date, there are no available standardized guidelines for training. LUS is also limited by its ability to evaluate superficial structures and could miss deep or central pathology. More data is needed for routine recommendation for LUS’ use in neonatal resuscitation. Despite its limitations, neonatal LUS has gained popularity as the extension of the stethoscope and is the standard of care for the assessment of neonatal respiratory distress in some units.

## Data Availability

Not applicable.

## Supplementary Materials

The following are available online at https://www.mdpi.com/article/10.3390/children8121098/s1, Video 1A: Lung sliding in infant with RDS, S1B: Lung sliding in infant without respiratory distress; Video 2: Large pleural effusion; Video 3: Atelectasis.
