# Supplementary figures and images for: Lung Ultrasound in the Neonatal Intensive Care Unit: Does It Impact Clinical Care?
Source: Children (Basel). 2021 Nov 29;8(12):1098. doi: 10.3390/children8121098 (PMC8700415; doi:10.3390/children8121098)

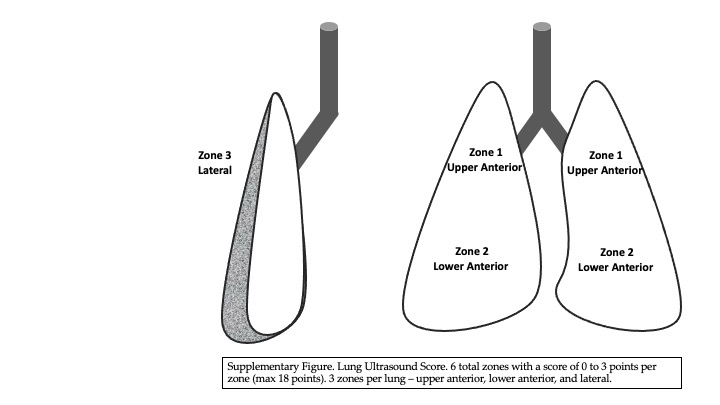

Supplement: Supplementary file 1 [file children-08-01098-s001.zip › Slide1.jpeg]

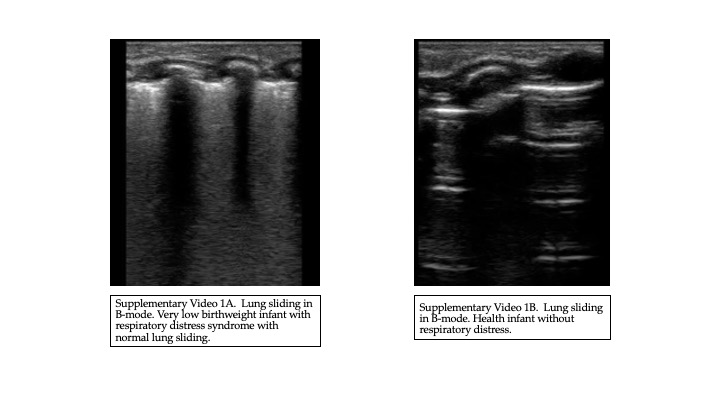

Supplement: Supplementary file 1 [file children-08-01098-s001.zip › Slide2.jpeg]

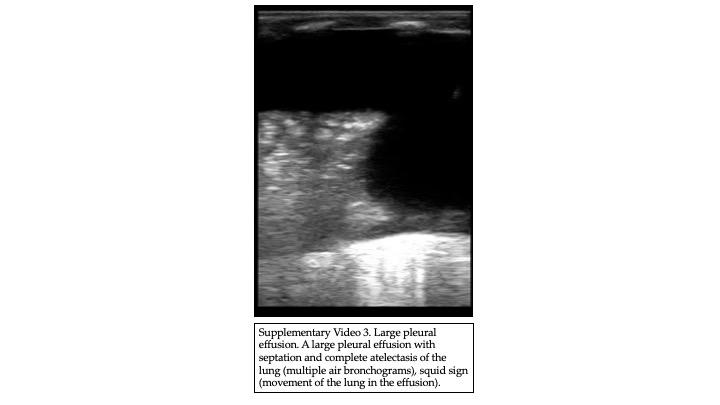

Supplement: Supplementary file 1 [file children-08-01098-s001.zip › Slide3.jpeg]

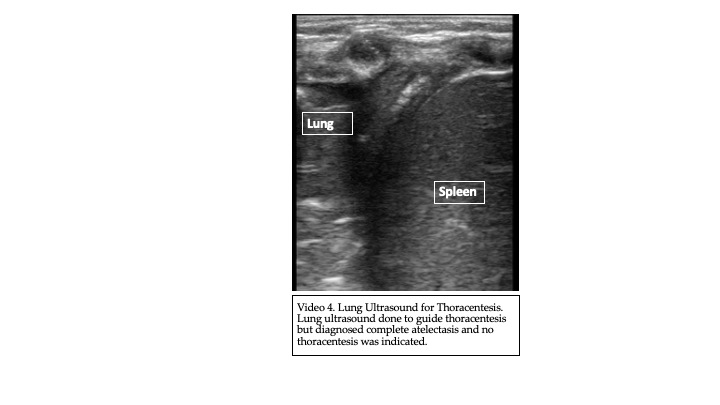

Supplement: Supplementary file 1 [file children-08-01098-s001.zip › Slide4.jpeg]
